# Lack of detectable HPV18 antibodies in 14% of quadrivalent vaccinees in a longitudinal cohort study

**DOI:** 10.1038/s41541-024-00941-w

**Published:** 2024-08-13

**Authors:** Penelope Gray, Filipe Colaço Mariz, Carina Eklund, Tiina Eriksson, Helena Faust, Hanna Kann, Martin Müller, Jorma Paavonen, Ville N. Pimenoff, Peter Sehr, Heljä-Marja Surcel, Joakim Dillner, Tim Waterboer, Matti Lehtinen

**Affiliations:** 1 Center for Cervical Cancer Elimination, Department of Clinical Science, Intervention and Technology, Karolinska Institutet, Stockholm, Sweden; 2 Tumorvirus-Specific Vaccination Strategies, Deutsches Krebsforschungszentrum (DKFZ), Im Neuenheimer Feld 242, 69120 Heidelberg, Germany; 3 Tampere University, Faculty of Medicine and Health Technology, Tampere, Finland; 4 Wellbeing services county of Pirkanmaa, PIRHA, Tays Research Services, Tampere, Finland; 5 Medical Products Agency Läkemedelsverket, Uppsala, Sweden; 6 Department of Microbiology and Immunology, University of Gothenburg, Gothenburg, Sweden; 7 Medical Faculty, University of Helsinki, Helsinki, Finland; 8 Unit of Population Health, Faculty of Medicine, University of Oulu, Oulu, Finland; 9 Biobank Borealis of Northern Finland, University of Oulu, Oulu, Finland; 10 EMBL-DKFZ Chemical Biology Core Facility, European Molecular Biology Laboratory (EMBL), Heidelberg, Germany; 11 Infections and Cancer Epidemiology, Deutsches Krebsforschungszentrum (DKFZ), Im Neuenheimer Feld 242, 69120 Heidelberg, Germany

**Keywords:** Epidemiology, Protein vaccines

## Abstract

Although HPV vaccines are highly efficacious, a notable proportion of quadrivalent vaccinees are HPV18 seronegative post-vaccination. We have investigated this findings’ validity by comparing vaccine-induced antibody responses using two different immunoassays. 6558 16–17-year-old females participated in the FUTURE II (NCT00092534) and PATRICIA (NCT00122681) trials in 2002–2004. Both the quadrivalent and bivalent vaccine recipients (QVR and BVR) received three doses. Twelve-year follow-up for 648 vaccinees was conducted by the Finnish Maternity Cohort. The presence of neutralising and binding HPV antibodies was analysed via HPV pseudovirion-based neutralisation and pseudovirion-binding assays. Four percent and 14.3% of the QVRs were seronegative for neutralising and binding antibodies to HPV16 and HPV18, respectively. No BVRs were HPV16/18 seronegative post-vaccination. The antibody titres were strongly correlated between the assays, Pearson’s correlation coefficient, *r*_[HPV16]_ = 0.92 and 0.85, and *r*_[HPV18]_ = 0.91 and 0.86 among the QVRs and BVRs respectively. Fourteen percent of QVRs lacked detectable HPV18 antibodies in long-term follow-up.

## Introduction

Quadrivalent (HPV6/11/16/18) and bivalent (HPV16/18) human papillomavirus (HPV) vaccines licensed 15 years ago to prevent HPV infection and related malignancies comprise virus-like particles (VLP) of the major capsid protein L1^1^. Several immunogenicity and efficacy trials have been conducted for both the bivalent and quadrivalent HPV vaccines, demonstrating not only long-term safety and immunogenicity^2–6^, but also robust protection against transient^7^ and persistent HPV16/18 infections^8–10^, all grades of cervical intraepithelial neoplasia (CIN) caused by HPV types 16 and 18^9,10^ and cervical cancer^11,12^. More recently, a nonavalent vaccine (HPV6/11/16/18/31/33/45/52/58) has been licensed and widely implemented^13^, with several more newly developed biosimilar vaccines also undergoing liscensure^14,15^.

Evidence obtained in animal challenge models demonstrated that protective antibodies induced via the L1 VLP vaccination can be passively transferred in serum from vaccinated to naïve animals, being able to reach mucosal sites and mediate virus neutralization^16^. High levels of neutralising antibodies are elicited by the HPV vaccines^6^. Both vaccine HPV type and restricted cross-reactive HPV type protection observed in clinical trials reflect the extent of antibody-induced neutralisation and cross-neutralisation^1,6^. Furthermore, the aggregated vaccine-induced neutralising and cross-neutralising antibody levels significantly correlated with efficacy against persistent HPV infection^6^. However, systematic head-to-head immunogenicity comparisons between the bivalent, quadrivalent and nonavalent vaccines are limited in number^2,3,17–19^.

Different independent, investigator-initiated studies showed that the bivalent and quadrivalent vaccines induce seroconversion of binding and neutralising anti-HPV16/18 L1 antibodies in up to 100% of recipients at the peak-immunogenicity interval (7-month after the 3rd dose), but at very different antibody levels^19–21^. Levels of antibodies to HPV induced by both the bivalent and quadrivalent vaccines have been shown to reach a plateau well above the natural seropositivity level among the majority of vaccinated individuals within 4 years post-vaccination^2,19,22^. Furthermore, detected long-term vaccine-induced antibody levels have been shown to remain sustainable for up to 12 years post-vaccination^3,6,22,23^. However, HPV18 antibody response has been undetectable in long-term follow-up among a minority of individuals vaccinated with the quadrivalent vaccine^3,5,6,19,24^.

Understanding the immunobiology of vaccine-induced antibody response over time through long-term immunogenicity studies has become increasingly important considering the global aim set by the WHO toward elimination of cervical cancer and reduced dose HPV immunization programmes^25,26^. In the present work, we further investigated the nature and consistency of HPV16/18 sero-responses in a head-to-head comparison of two cohorts of HPV vaccinated women by two different well-established assays: the multiplexed pseudovirion-binding neutralisation assay (HT-PBNA) and the heparin bound HPV-pseudovirion based Luminex assay.

## Results

**Fig. 1:**
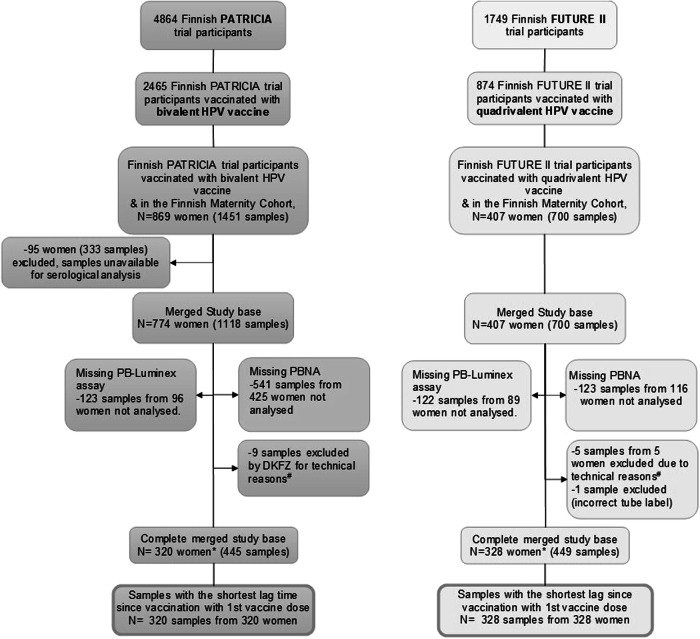
Flow chart of the study population. *All women have received 3 doses of their allotted HPV vaccine. #Fourteen sera had inconsistent results across the HT-PBNA and the GST-L1 or VLP-Luminex assays carried out in parallel, which we interpreted as the result of a pipetting error. DKFZ=German Cancer Research Center.

In 2002-2003 and 2004-2005, respectively, 1749 Finnish females participated in the FUTURE II trial of the quadrivalent vaccine and 4864 Finnish females participated in the PATRICIA trial of the bivalent vaccine in late adolescence^27,28^. From those women, 2465 females received the bivalent HPV16/18 vaccine, and 874 females received the quadrivalent HPV6/11/16/18 vaccine, out of which 869 and 407 women respectively donated at least one sample to the FMC Serum Bank by the end of 2016. A total of 1118 samples from 774 BVRs and 700 samples from 407 QVRs were available for serological analyses, from which 445 samples from the BVRs and 449 samples from the QVRs were selected for analyses of both binding and neutralising antibodies. Only the samples with the shortest lag time since vaccination to blood sampling were included for each woman, leading to a total of 320 and 328 samples being included from the 320 BVRs and 328 QVRs, respectively (Fig. 1).

The majority of the participants were aged 22–29 years old when donating their serum sample with the shortest lag time since vaccination, with the mean age being 25.4 years-old among the QVRs and 25.6 years-old among the BVRs (Table 1). The mean lag time between the time of receiving the first dose of their allotted vaccine and the time of sample donation was also similar between the two cohorts, measuring as 8.9 and 9.1 years among the QVRs and BVRs, respectively (Table 1). HPV16 and 18 antibody levels were relatively stable over time among both the BVRs and the QVRs (Supplementary Figs 1 and 2).

**Table 1:** Descriptive characteristics of the study population of female vaccine recipients stratified by the HPV vaccine received

	Quadrivalent vaccine recipients, *N* = 328	Bivalent vaccine recipients, *N* = 320
*Characteristic*	*n*/*N* (%)	*n*/*N* (%)
*Birth year cohort*		
*1984*	28/328 (8.5)	0/320 (0.0)
*1985*	157/328 (47.9)	0/320 (0.0)
*1986*	140/328 (42.7)	2/320 (0.6)
*1987*	3/328 (0.9)	185/320 (57.8)
*1988*	0/328 (0.0)	133/320 (41.6)
*Lag time between 1st vaccine dose and sample donation (years)*		
*1-2*	7/328 (2.1)	0/320 (0.0)
*3-4*	2/328 (0.6)	0/320 (0.0)
*5-6*	40/328 (12.2)	26/320 (8.1)
*7-8*	114/328 (34.8)	128/320 (40.0)
*9-10*	114/328 (34.8)	136/320 (42.5)
*11-12*	51/328 (15.5)	30/320 (9.4)
*Age at 1st vaccination dose (years)*		
*15*	6/328 (1.8)	0/320 (0.0)
*16*	147/328 (44.8)	161/320 (50.3)
*17*	155/328 (47.3)	152/320 (47.5)
*18*	20/328 (6.1)	7/320 (2.2)
*Age at time of sample donation (years)*		
*18-19*	3/328 (0.9)	1/320 (0.3)
*20-21*	6/328 (1.8)	0/320 (0.0)
*22-23*	44/328 (13.4)	34/320 (10.6)
*24-25*	108/328 (32.9)	118/320 (36.9)
*26-27*	114/328 (34.8)	126/320 (39.4)
*28-29*	53/328 (16.2)	41/320 (12.8)
	Mean (standard deviation)	
*Age when vaccinated (years)*	17 (0.6)	17 (0.5)
*Age at sample donation (years)*	25.4 (2.0)	25.6 (1.6)
*Lag time between 1st vaccine dose and sample donation (years)*	8.86 (1.9)	9.07 (1.4)

Among the QVRs, 4.0% (13/328) were seronegative for HPV16 neutralising antibodies as measured by the PBNA, while 4.6% (15/328) were seronegative for HPV16 total binding antibodies (Table 2). Altogether, 4.0% (13/328) were seronegative for both HPV16 neutralising and total binding antibodies (Cohen’s Kappa coefficient equal to 0.93, 95% confidence intervals, CI, 0.82–1.00) (Table 2). In comparison, no BVRs were seronegative to HPV16 neutralising or binding antibodies.

**Table 2:** HPV16 seronegativity among the bivalent vaccine recipients (BVR) and quadrivalent vaccine recipients (QVR) (including only the samples with the shortest lag)

	HPV16 seronegativity among trial participants 1–12 years post-vaccination with 3 doses, *n*/*N* (%)			
	Neutralising antibodies (PBNA)	Total binding antibodies (Luminex)	PBNA & Luminex	Kappa, κ
*All vaccine recipients*	13/648 (2.00%)	15/648 (2.31%)	13/648 (2.00%)	0.93 (0.83–1.00)
*Quadrivalent vaccine recipients*	13/328 (3.96%)	15/328 (4.57%)	13/328 (3.96%)	0.93 (0.82–1.00)
*Bivalent vaccine recipients*	0/320 (0.00%)	0/320 (0.00%)	0/320 (0.00%)	-

Cohen’s Kappa coefficient comparing the results from the PBNA to that from the Luminex immunoassay. *HPV16 seronegative as measured both with the PBNA and the Luminex immunoassay.

When assessing the immune response to HPV18, among the QVRs, 23.2% (76/328) were measured as seronegative for binding HPV18 antibodies, while 14.6% (48/328) of the women were seronegative for HPV18 neutralising antibodies (Table 3). When further comparing the results, 14.3% (47/328) were HPV18 seronegative for both neutralising and total binding antibodies (Kappa coefficient equal to 0.71, 95% CI 0.60–0.81), and 8.8% (29/328) lacked total HPV pseudovirion binding antibodies despite having detectable neutralising HPV18 antibodies (Table 3). When the samples discordant for HPV18 (neutralising antibody seropositive and binding antibody seronegative) were reanalysed, 100% were seropositive for HPV18 total binding antibodies, but with very low antibody titres (geometric mean titre = 0.52 IU). Among the BVRs, no participants were seronegative for HPV18 neutralising antibodies and only 0.63% (2/320) were seronegative for binding HPV18 antibodies (Table 3).

**Table 3:** HPV18 seronegativity among the bivalent vaccine recipients (BVR) and quadrivalent vaccine recipients (QVR) (including only the samples with the shortest lag)

	HPV 18 seronegativity among trial participants 1–12 years post-vaccination with 3 doses			
	Neutralising antibodies (PBNA)	Total binding antibodies (Luminex)	PBNA & Luminex^a^	Kappa, κ
*All vaccine recipients*	48/648 (7.41%)	78/648 (12.0%)	47/648 (7.25%)	0.72 (0.63–0.81)
*Quadrivalent vaccine recipients*	48/328 (14.6%)	76/328 (23.2%)	47/328 (14.3%)	0.71 (0.60–0.81)
*Bivalent vaccine recipients*	0/320 (0.00%)	2/320 (0.63%)	0/320 (0.00%)	-

^a^HPV18 seronegative as measured both with the PBNA and the Luminex immunoassay.

Cohen’s Kappa coefficient comparing the results from the PBNA to that from the Luminex immunoassay.

**Fig. 2:**
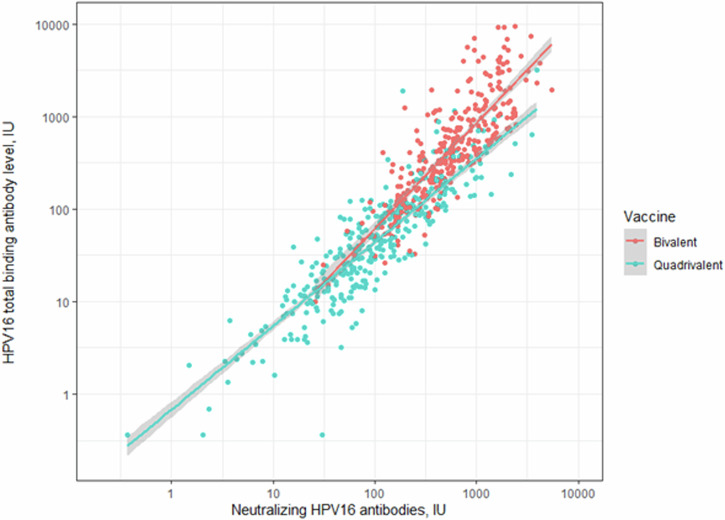
Scatterplot of HPV16 neutralising antibody levels versus total binding antibody levels (in international units, IU). Pearson’s correlation coefficient (among all vaccinated participants), *ρ* = 0.92 (95% confidence intervals 0.91–0.93), *ρ* (among BVRs) = 0.85 (0.82–0.88), and *ρ* (among QVRs) = 0.92 (0.90–0.93).

**Fig. 3:**
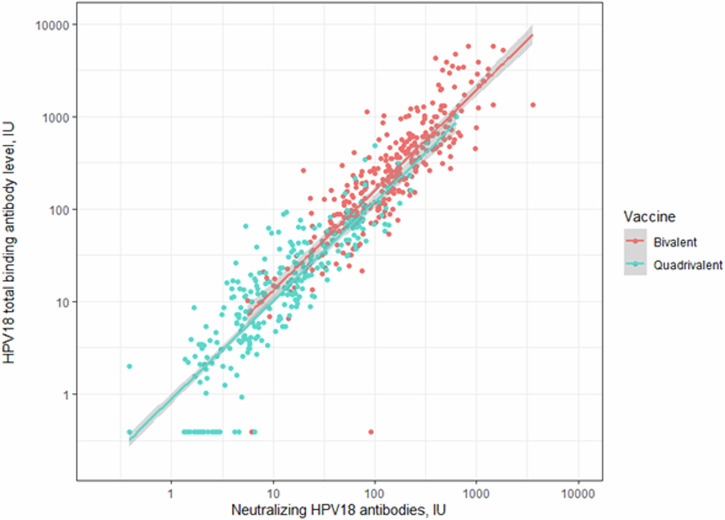
Scatterplot of HPV18 neutralising antibody titres versus total binding antibody level. Pearson’s correlation coefficient (among all vaccinated participants), *ρ* = 0.95 (95% confidence intervals 0.94–0.96), *ρ* (among BVR) = 0.86 (0.83-0.89) and *ρ* (among QVR) = 0.91 (0.89–0.93).

Among both the QVR and the BVR both the HPV16 neutralising antibody titre and HPV18 neutralising antibody titres were strongly correlated with the total binding antibody titre of the same HPV type irrespective of the vaccine (ρ_[HPV16, BVR]_ = 0.85 [0.82–0.88], ρ_[HPV16, QVR]_ = 0.92 [0.90–0.93], ρ_[HPV18, BVR]_ = 0.86 [0.83–0.89] and ρ_[HPV18, QVR]_ = 0.91 [0.89–0.93]) (Figs. 2 and 3). When the HPV16 neutralising antibody titre was assessed in relation to the HPV18 neutralising antibody titre among the vaccine recipients, the HPV16 neutralising antibody titre was strongly correlated to the individuals HPV18 neutralising antibody titre among both the QVRs and the BVRs (ρ_[QVR]_ = 0.67 [0.60–0.72], ρ_[BVR]_ = 0.68 [0.62–0.74]) (Supplementary Fig. 3). Among the vaccine recipients whose HPV18 neutralising antibody levels were within the lowest antibody quartile, the correlation between the HPV16 and HPV18 neutralising antibody titre was moderate irrespective of the vaccine received (*r*_s[BVR]_ = 0.31 [95% CI, 0.09–0.51] and *r*_*s*[QVR]_ = 0.41 [0.19–0.58]).

When assessing if being HPV16 or 18 seronegative impacted the risk of being seronegative to the other vaccine targeted HPV types the following was found among the quadrivalent vaccine recipients: the risk of being seronegative for HPV16 neutralising antibodies was found to be 70-fold higher (95% CI 9.3–530) among those HPV18 seronegative for neutralising antibodies as compared to those seropositive. Similarly, the risk of being seronegative for HPV18 neutralising antibodies was 8.1-fold higher (95% CI 5.7–11.4) among QVRs seronegative for HPV16 neutralising antibodies in comparison to those seropositive (Tables 4 and 5). For phylogenetically related HPV types, seronegativity against either HPV16 or 18 were consistently associated with an increased risk of being seronegative to neutralising antibodies against HPV31, 33, 45, 52 or 58 also (Tables 4 and 5).

**Table 4:** Prevalence ratio of HPV16, 31, 33, 45, 52 and 58 seronegativity comparing HPV18 seronegative to HPV18 seropositive among quadrivalent vaccine recipients

	Prevalence ratio (95% confidence intervals) of seronegativity comparing the HPV18 seronegative to the HPV18 seropositive women
*HPV type*	Neutralizing HPV18 antibody seronegative vs. seropositive
*HPV16*	70.0 (9.32–526)
*HPV45*	1.16 (1.07–1.26)
*HPV31*	1.60 (1.31–1.97)
*HPV33*	1.35 (1.15–1.60)
*HPV52*	1.86 (1.40–2.47)
*HPV58*	1.34 (1.15–1.57)
*Alpha 9 Clade seronegative to any (16/31/33/52/58)*	1.24 (1.17–1.31)
*Alpha 9 Clade seronegative to all (16/31/33/52/58)*	ne

*HPV* seronegativity is measured via type specific neutralizing or cross-neutralizing antibody seronegativity. *ne* not possible to estimate.

**Table 5:** Prevalence ratio of HPV18, 31, 33, 45, 52 and 58 seronegativity comparing HPV16 seronegative to HPV16 seropositive among quadrivalent vaccine recipients

	Prevalence Ratio (95% confidence intervals) of seronegativity comparing the HPV16 seronegative to the HPV16 seropositive women
*HPV Type*	Neutralising HPV16 antibody seronegative vs. seropositive
*HPV18*	8.08 (5.72–11.4)
*HPV45*	1.19 (1.14–1.25)
*HPV31*	1.88 (1.55–2.28)
*HPV33*	1.36 (1.06–1.74)
*HPV52*	2.20 (1.58–3.08)
*HPV58*	1.44 (1.21–1.72)
*Alpha 7 clade seronegative to any (18/45)*	1.18 (1.13–1.24)
*Alpha 7 clade seronegative to all (18/45)*	8.55 (6.00–12.2)
*Alpha 9 Clade seronegative to any (31/33/52/58)*	1.24 (1.17–1.31)
*Alpha 9 Clade seronegative to all (31/33/52/58)*	5.14 (3.19–8.30)

*HPV* seronegativity is measured via type specific neutralising or cross-neutralising antibody seronegativity.

When we assessed the seropositivity to HPV6 neutralising antibodies stratified by HPV18 seropositivity, among the QVRs 95.8% (46/48) of the HPV18 seronegative women were seropositive for HPV6 neutralising antibodies, and 99.6% (279/280) of the HPV18 seropositive women were seropositive for HPV6. Conversely, 88.8% of BVRs (284/320) were seropositive for HPV6. The HPV6 geometric mean titre was also consistently higher among the QVRs than the BVRs regardless of HPV18 seropositivity or HPV18 antibody quartile (Table 6).

**Table 6:** HPV6 and HPV16 neutralising antibody geometric mean titre stratified by HPV18 neutralising antibody titre quartile and the vaccine received (either [a] the quadrivalent or [b] bivalent HPV vaccine)

	a) Quadrivalent vaccine recipients					
	HPV18 antibody titre					
	HPV18 Seronegative	HPV18 Seropositive				
	All	Q1	Q2	Q3	Q4	All
HPV6 GMT^a^ (EC50)	700	2138	3651	5477	11683	4730
HPV16 GMT^b^ (IU)	25	47.9	80.9	157.4	296	115

	b) Bivalent vaccine recipients					
	HPV18 antibody titre					
	HPV18 Seronegative	HPV18 Seropositive				
	All	Q1	Q2	Q3	Q4	All
HPV6 GMT^a^ (EC50)	na	152	129	182	218	168
HPV16 GMT^b^ (IU)	na	236	447	698	1367	564

^a^Among those HPV6 seropositive. ^b^Among those HPV16 seropositive.

## Discussion

We compared the HPV16 and HPV18 total binding antibody response to neutralising antibody response among HPV vaccinated participants of two phase 3 clinical trials on the efficacy of the bivalent and quadrivalent vaccines. One in 7 women vaccinated with the quadrivalent vaccine had no measurable neutralising nor total binding antibodies to HPV18. In comparison, all of the women vaccinated with the bivalent vaccine had measurable levels of anti-HPV16 and 18 antibodies. The HPV16 or 18 type-specific neutralising antibody levels were found to be strongly correlated with the total binding antibody levels for the same HPV type irrespective of the vaccine received. Further to this, we found that among the QVRs the risk of being HPV16 seronegative was greatly increased if a woman was seronegative for HPV18 (and vice versa). Among all the HPV vaccinated women, the HPV16 neutralising antibody titre was strongly correlated with the HPV18 antibody titres. The lack of detectable HPV18 antibody was not accompanied by HPV6/HPV16 antibody seronegativity, which suggests that the QVRs were fully vaccinated, and the lower HPV18 antibody responsiveness was not due to any issues at the vaccination site.

Early follow-up studies of the two efficacy licensing trials demonstrated that vaccine-induced antibody levels to HPV16/18 peaked 7 months post-vaccination. Earlier, in 40% of the QVRs HPV18 antibody levels decayed to undetectable levels at 4 years post-vaccination^29^, which was initially speculated to result from an imprecision of the utilized serological assay (competitive Luminex Immuno-assay, as comprehensively reviewed by Schiller et al, 2012 and Stanley & Pinto, 2012)^1,30^. Our follow-up of trial participants reported that variable proportions of QVRs had no detectable HPV18 antibodies 2–12 years post-vaccination when tested by the neutralisation assay^6^ and a HPV pseudovirion based Luminex assay^3^ independently. Herein, the strong agreement in HPV16/18 serostatus between the two assays and the strong positive correlation between the neutralising and total binding antibodies suggests that the finding of 14% of quadrivalent vaccinees being seronegative in long term follow-up is unlikely to be due to laboratory error but a true biological phenomenon.

Reasons for the distinct antibody responses induced by these two HPV vaccines are under debate. It has been postulated that AS04, the adjuvant used in the bivalent vaccine, affords stronger immune responses than the AAHS (aluminium salt) adjuvant used in the quadrivalent vaccine, and may explain the higher HPV16 and 18 antibody levels among BVRs^1,3^. On the other hand, the vaccine induced HPV16 antibody responses have universally been found to be greater than the HPV18 antibody responses in BVRs^2,3,19^, despite the equal amounts of HPV16/18 L1 proteins (20 micrograms) per dose in the bivalent vaccine. The markedly lower HPV18 neutralising/binding antibody levels observed in the QVRs, despite the vaccine being formulated with comparable amounts (20 µg) of HPV6/18 L1 proteins, further support the notion that HPV18 VLPs are comparatively less immunogenic than its HPV6 VLP counterparts, even if this has never been experimentally demonstrated. Previous studies have reported that different adjuvants may have a role in mitigating immunodominance, although whether ASO4 of the bivalent vaccine is better at mitigating immunodominance is unclear^31^. In addition, the different expression systems of the VLPs may have possibly resulted in qualitative differences in the VLPs of the two vaccines. Finally, there are also in silico evidence that amino acid variants of the L1 protein of the major oncogenic HPV types may determine differing VLP immunogenicity^32^, which have not been validated experimentally yet. According to these computational predictions, however, these differences are more likely impacting HPV16 VLP immunogenicity but not HPV18.

Interestingly, 8% of the QVRs were found to be seronegative for HPV18 binding antibodies whilst seropositive for neutralising antibodies, albeit with a low geometric mean antibody titre. Hypothetically it may have been due to the presence of HPV18-neutralising IgA in the serum not measured by the binding assay but measurable via the neutralisation assay^33^. Upon re-analysis of these discordant samples for total binding antibodies, they were all found to be HPV18 seropositive, but with an extremely low geometric mean binding antibody titre just above the cut-off for seropositivity. We think that the previous discordancy of these samples may have arisen due to the lack of an internationally standardised cut-off (in international units) for HPV16 and 18 seropositivity and/or intrinsic assay variability at extremely low antibody titres.

This study is strengthened by the comparison of the results of two immunoassays conducted in two independent laboratories, and the reporting of the antibody titres in international units which allows for the direct comparison of the quantitative results.

Our study likely has high generalisability to women vaccinated in late adolescence. However, it may be limited in its transportability to populations vaccinated at younger ages. It is well established that HPV vaccination in childhood induces a stronger immune response compared to vaccination in late adolescence^34^. Our study is also not generalisable to immunocompromised populations, for whom the immunogenicity is poorer^35^. Crucially, all the participants received three vaccine doses, therefore caution should be taken when generalising our results to populations vaccinated with only one dose. Furthermore, in studies reporting the long-term immunogenicity of the quadrivalent vaccine after reduced dosing schedules, the long term HPV18 neutralising antibody seropositivity was reported to be as low as 48% after one dose^36^.

Despite reports of quadrivalent vaccinees having no detectable HPV18 antibodies several years post-vaccination, the corresponding vaccine efficacy against persistent infection and associated disease is high, 92% and 100%, respectively^8^. Therefore, given the lack of clinical endpoints in our study it is unknown whether our reported lack of detectable HPV16/18 antibodies translates into increased risk of HPV infection and associated disease. If seronegativity among quadrivalent vaccinees does increase the risk of HPV infection, these risks may be mitigated in countries with high HPV vaccination coverage due to herd protection. Furthermore, although this study evaluates the presence of both binding and functional vaccine induced antibodies, it does not measure other mechanisms possibly driving protection in vivo such as Fc-mediated antibody effector functions^37,38^. Finally, we did not analyse samples from the peak immunogenicity interval, thus we cannot conclude whether the HPV16/18 seronegative women in our study ever seroconverted post-vaccination.

In conclusion, 14% of women vaccinated with three doses of the quadrivalent HPV vaccine in late adolescence had no measurable HPV18 antibodies 2–12 years afterwards, while 4% had no detectable HPV16 antibodies. In comparison, women who had been vaccinated with the bivalent vaccine had measurable HPV16 and 18 antibodies 2–12 years afterwards. A lack of HPV18 antibodies was strongly associated with the risk of being seronegative for HPV16 antibodies and vice versa among the quadrivalent vaccine recipients, as were the levels of these antibodies. Further surveillance of seronegative women after quadrivalent vaccination is crucial.

## Methods

### Study design

In this study we have conducted a long-term follow-up among the Finnish participants randomised to the intervention arms of two international phase III HPV vaccination trials, the FUTURE trial (NCT00092534) and the PATRICIA trial (NCT00122681)^27,28^. The FUTURE trial was initiated in 2002. A total of 1745 Finnish females aged 16-17 years old from the 1984–87 birth cohorts were enrolled and individually randomised (using a computer-generated randomized allocation schedule) to receive either the quadrivalent HPV6/11/16/18 vaccine, Gardasil, or a placebo in an allocation ratio of 1:1^27^. The participants were then vaccinated with either the quadrivalent vaccine or the placebo, at 0, 2 and 6 months, in a double blinded manner^27^.

The PATRICIA trial commenced in 2004/5. A total of 4808 Finnish females aged 16-17 years old from the 1986–88 birth cohorts were enrolled and individually randomised (using an internet-based centralised randomisation system) to receive either the bivalent HPV16/18 vaccine, Cervarix, or the hepatitis A virus (HAV) vaccine^28^. The participants were randomised in an allocation ratio of 1:1 and received three doses of the HPV or HAV vaccine in a 0-, 1- and 6-month schedule and also in a double blinded manner^28^.

The Finnish participants of these two trials who were randomised to receive the HPV vaccine form two parallel cohorts of HPV vaccinated women. The study population of this current study are sub-cohorts nested within these two parallel cohorts and comprise of the HPV vaccinated participants who also donated serum samples to the Finnish Maternity Cohort by the end of 2016. The Finnish Maternity Cohort (FMC) is a large population representative biobank containing serum samples^28^. These serum samples are comprised of samples given by pregnant women when attending the maternity clinic to give a blood sample for routine congenital screening between 1983 until the end of 2016^39^. Approximately 96% of all pregnant women have consented to have their sample stored in the biobank, resulting in approximately 2,000,000 samples from 1,000,000 women comprising the FMC^39^.

In spring 2014, the Finnish HPV vaccination registry (of women vaccinated via trials) was linked with the FMC to identify all the HPV vaccinated trial participants who had serum samples stored in the Finnish Maternity biobank. Linkage was conducted using the participants unique Finnish personal identification number (given to all Finnish residents at birth, or entry to the country). A further linkage was done in autumn 2016 to identify all women who had donated samples since. All of the identified FMC samples from these women were then extracted from the biobanks’ −20 °C freezers and aliquoted for the purpose of serological testing.

From these women and samples which were identified, we applied further inclusion criteria to be included in this study. For the samples from the participants who received the bivalent vaccine (BVRs) we used a random number generator to select a random selection of samples which were closest aligned to the samples from the quadrivalent vaccine recipients (QVRs) in terms of follow-up time and number of pregnancies^3^. In this study we further included only women who had received three doses of their allotted HPV vaccine, and only the serum samples donated by the participants with the shortest lag time between the date of first vaccination and serum sampling (in the case of multiple samples from one woman). These serum samples have previously been analysed by two different laboratories using two different laboratory methods (Luminex immunoassay and HT-PBNA) (Fig. 1)^3,6^.

### Inclusion and ethics

The PATRICIA trial (ClinicalTrials.gov, NCT00122681, registered on the 2005-07-27), the FUTURE II trial (ClinicalTrials.gov, NCT00092534, registered on the 2006-01-12) and the long-term follow-up of the trial participants were approved by the Finnish National Review Board (ETENE/Tukija) in 2004 and 2002 respectively (HPV008 17/04/04, 5.4.2004 and 015-00 58.04.02, 10.6.2002). The women participating in the Finnish part of the PATRICIA and FUTURE II trials gave informed consent to participate in the original trials and for their long-term follow-up in the Finnish health registries including the FMC serum bank. The study design and the selection of FMC biobanked serum samples were approved by the responsible Biobank’s Scientific Committee. The study was conducted in accordance with the Declaration of Helsinki.

### Laboratory methods

#### Heparin bound HPV pseudovirion-based Luminex immunoassay

The serum samples from the study population were analysed for the presence and concentration of total binding antibodies to HPV6, 11, 16, 18, 31, 33, 35, 39, 45, 51, 52, 56, 58, 59, 68 and 73 using a multiplexed heparin-bound HPV pseudovirion based Luminex immunoassay^3,40^. The assay uses type specific L1/L2 HPV pseudovirions produced in mammalian cell culture and coupled to fluorescent Luminex beads coated in Heparin proteoglycan as described previously^40^. The serum samples were analysed in 5 dilutions (x50, x150, x450, x1350 and x4050)^3^. International standards for HPV16 and HPV18 were included in each assay run to be able to calculate the HPV16 and 18 antibody titres in international units^41,42^. To avoid differential assay drift by the type of vaccine received, samples from both of the parallel HPV vaccination cohorts were included in the same plates^3^. International units were calculated using the parallel line method and with reference factors of 10 and 16 for HPV16 and HPV18, respectively^43^.

#### High throughput pseudovirion-based neutralisation assay, HT-PBNA

Neutralising antibody levels to HPV types 6, 16, 18, 31, 33, 45, 52 and 58 were determined by a high-throughput neutralisation assay^6,44^. The assay employs HPV pseudovirion particles comprising of a Gaussia luciferase reporter plasmid encapsidated by the L1 and L2 proteins^44^. Following the production and purification from HEK293TT cells by ultra-centrifugation using Optiprep gradient, the pseudovirion particles are able to drive the transduction of the Gaussia luciferase into HeLaT reporter cells, which is quantified directly from the cell culture medium using the luminescent substrate colenterazine^44^. In the presence of neutralising antibodies, the Gaussia luciferase transduction is inhibited or reduced^44^. Serum samples were subjected to a serial dilution in seven steps, following a 3.33-fold increment, to reach a final dilution of 1:40 to 1:180,000 in the assay plate^6^. Neutralising antibody titres (EC50) were calculated as the serum dilutions in which a 50% inhibition of the luciferase activity was observed. EC50 values > 40 (corresponding to 1.3 and 1.1 IU/ml for HPV16 and HPV18, respectively) were defined as neutralising antibody positive^6^.

### Statistical methods

To evaluate possible differences in the characteristics between the two cohorts of HPV vaccinated individuals which might explain differences in the HPV16/18 seropositivity, we calculated the mean age in years when receiving the first dose of HPV vaccination, the mean age at FMC sample donation, and the mean time between the individual’s first vaccine dose and the serum sample donation (and corresponding standard deviations) stratified by the type of HPV vaccine received. The HPV16 and 18 antibody levels (neutralising and binding antibodies) were plotted over time since the women received their first dose of their respective vaccine, and locally weighted linear regression was used to evaluate the sustainability of the antibody levels over time stratified by the vaccine received. We then compared the occurrence of seronegativity for HPV16 total binding antibodies (measured via the Luminex assay) to seronegativity for HPV16 neutralising antibodies (measured via the PBNA), among all the participants and then stratified by the vaccine received (the bivalent or quadrivalent vaccine). To assess the agreement between the two assays, we then calculated Cohen’s Kappa coefficient, κ, and corresponding 95% confidence intervals for all participants, for the bivalent vaccine recipients and for the quadrivalent vaccine recipients. We then compared the occurrence of HPV18 seronegativity as measured via the two assays in an identical manner.

We then evaluated the risk of being seronegative for additional HPV types according to HPV16 or HPV18 serostatus among the quadrivalent vaccine recipients. This was assessed by estimating the relative risk (RR) (and 95% confidence intervals) of being HPV seronegative with i) type specific HPV16, 18, 45 31, 33, 52 and 58, ii) alpha clade 7 types combined (as defined as double seronegativity to HPV18 and 45) and iii) alpha clade 9 types combined (as defined as seronegativity to HPV16, 31, 33, 52 and 58), among the HPV16 or HPV18 seronegative compared to the HPV16 or HPV18 seropositive using a log binomial regression model. In the incidents where the log binomial model failed to converge, we calculated the RR and 95% confidence intervals using a modified Poisson regression model with robust standard errors, using R statistical software and the sandwich package (version 3.0-2).

We then assessed the geometric mean titre of neutralising antibodies to HPV6, an HPV type targeted by the quadrivalent vaccine, and HPV16, stratified by HPV18 neutralising antibody seropositivity and quartile and the type of vaccine received (into quadrivalent and bivalent vaccine recipients). Such analyses are informative for ruling out the possibility that weak anti-HPV18 antibody responses were due to failure to provide full HPV immunization, given the high proportion of QVRs which seroconverted to HPV6 and 16 and the high HPV6 and 16 antibody responses among the same.

Finally, we compared the correlation between the HPV16 total binding antibodies and the HPV16 neutralising antibodies (and similarly for HPV18 total binding and neutralising antibodies) by plotting the antibody titres in international units comparing the results of the two assays, and by calculating Pearson’s correlation coefficient and its associated 95% confidence intervals.

All statistical analyses were conducted using R statistical software version 4.2.1 (The R Foundation; https://www.r-project.org/).

### Sensitivity analyses

As a sensitivity analyses, all samples which were originally found to be HPV18 seronegative when analysed with the heparin bound HPV pseudovirion-based Luminex immunoassay but HPV18 seropositive when analysed with the PBNA, were retrieved and re-analysed using the Luminex immunoassay.

Furthermore, we also further examined the association between antibody response to one vaccine type to the vaccine response to another vaccine type, by plotting the HPV16 (and HPV6) neutralising antibody levels according to the HPV18 neutralising antibody levels, and subsequently calculating Pearson’s correlation coefficient of the 2 respective antibody levels (natural log transformed). We further evaluated the correlation between the HPV18 neutralising response and the HPV16 neutralising response by calculating Spearman’s correlation coefficient and 95% confidence intervals using bootstrapping stratified by vaccine among the vaccine recipients whose HPV18 antibody levels were in the lowest quartile.

### Supplementary information

Supplementary Information

## Data Availability

The data from this study is available upon reasonable request and ethical approval via FinnGen (https://www.finngen.fi).
