# Supplementary material for: Lack of detectable HPV18 antibodies in 14% of quadrivalent vaccinees in a longitudinal cohort study
Source: NPJ Vaccines. 2024 Aug 13;9:146. doi: 10.1038/s41541-024-00941-w (PMC11322158; doi:10.1038/s41541-024-00941-w)
Supplement: Supplementary file 1 — Supplementary Information [file 41541_2024_941_MOESM1_ESM.pdf]

Supplementary Material

Supplementary Figure 1: HPV16 i) neutralizing antibody level and ii) binding antibody level over time since HPV vaccination among women vaccinated with the quadrivalent or bivalent vaccine.

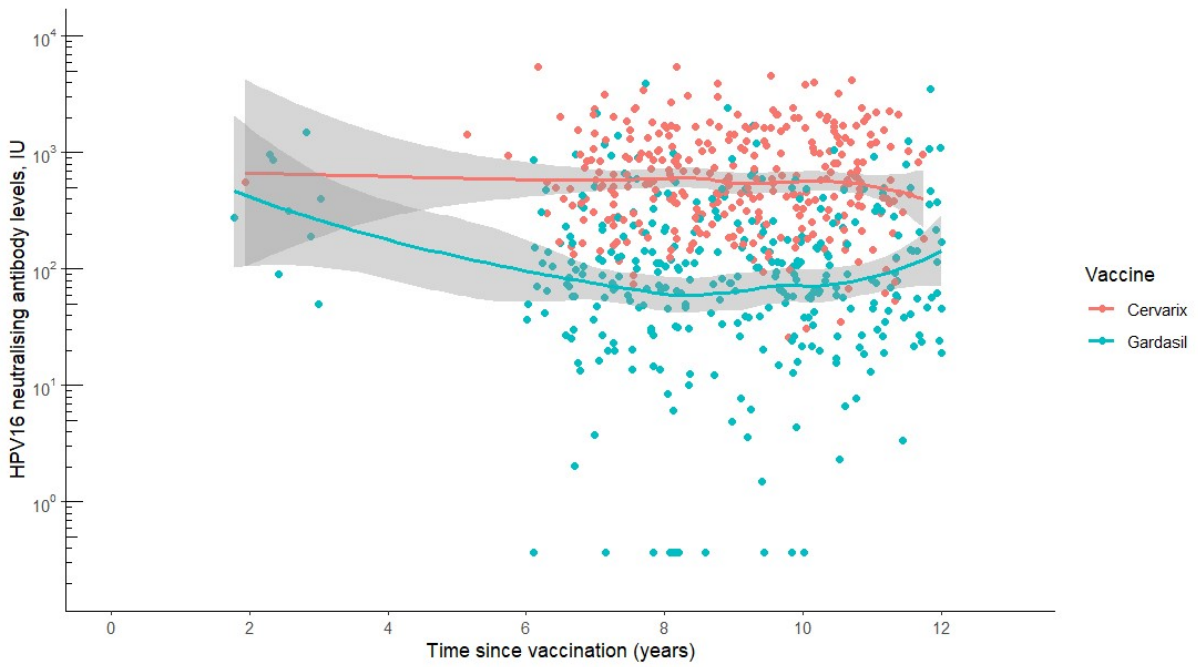

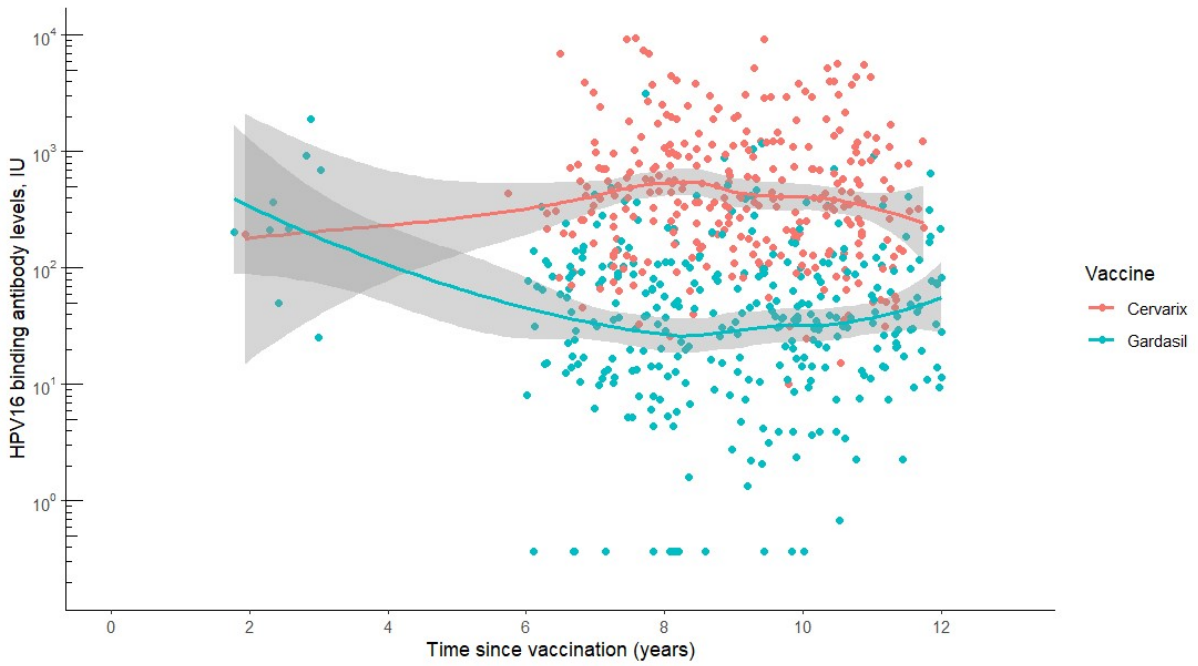

Supplementary Figure 2: HPV18 i) neutralizing antibody level and ii) binding antibody level over time since HPV vaccination among women vaccinated with the quadrivalent or bivalent vaccine.

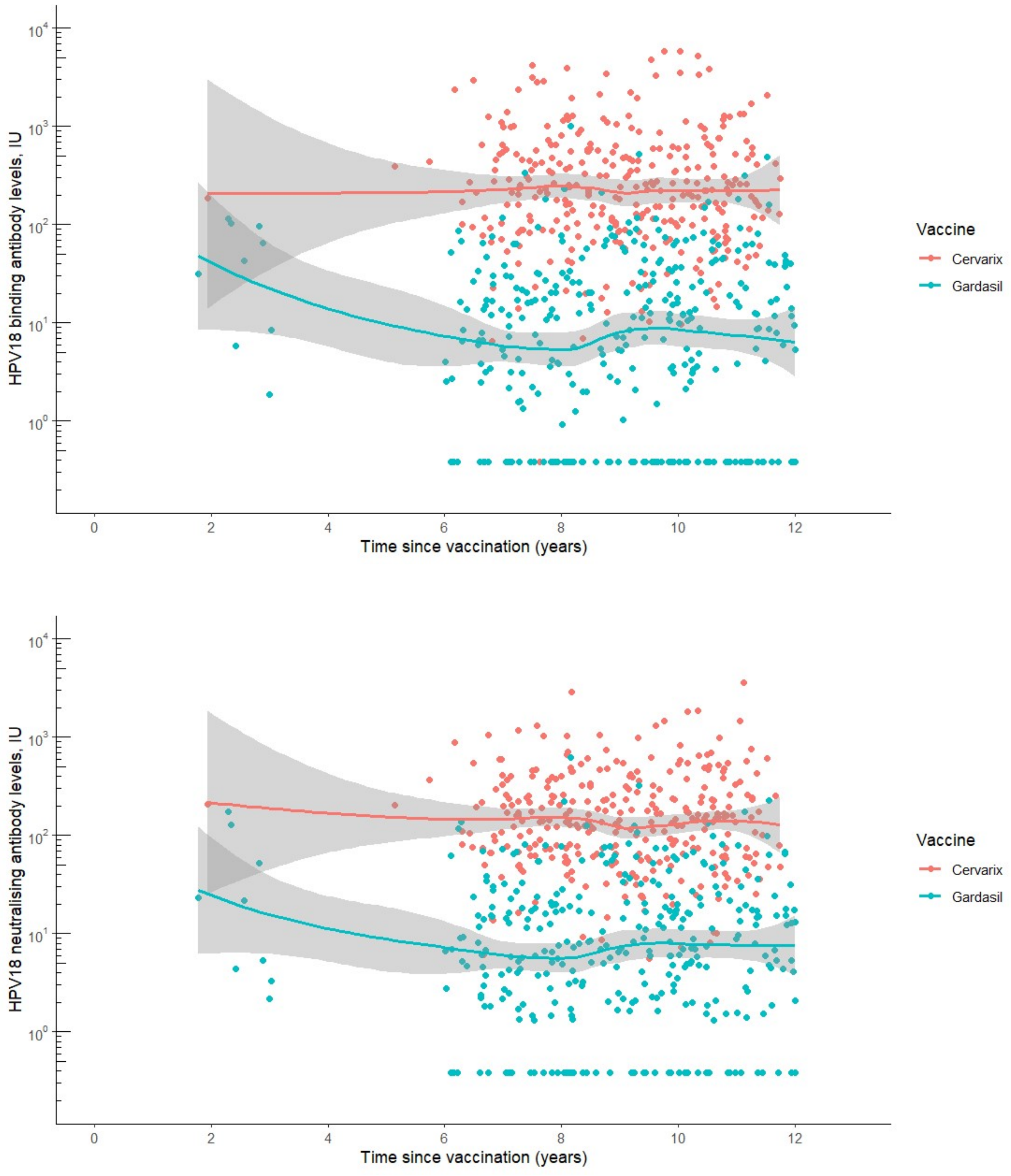

Supplementary Figure 3: Scatterplot of the neutralising HPV16 antibody titres versus neutralising HPV18 antibody titre among the vaccine recipients 2-12 years since vaccination with 3 doses of either the bivalent or quadrivalent vaccines. Pearsons's correlation coefficient, ρ (among quadrivalent vaccine recipients) = 0.67 (95% confidence intervals, CI, 0.60-0.72), ρ (among bivalent vaccine recipients) = 0.68 (95% confidence intervals, CI, 0.62-0.74).

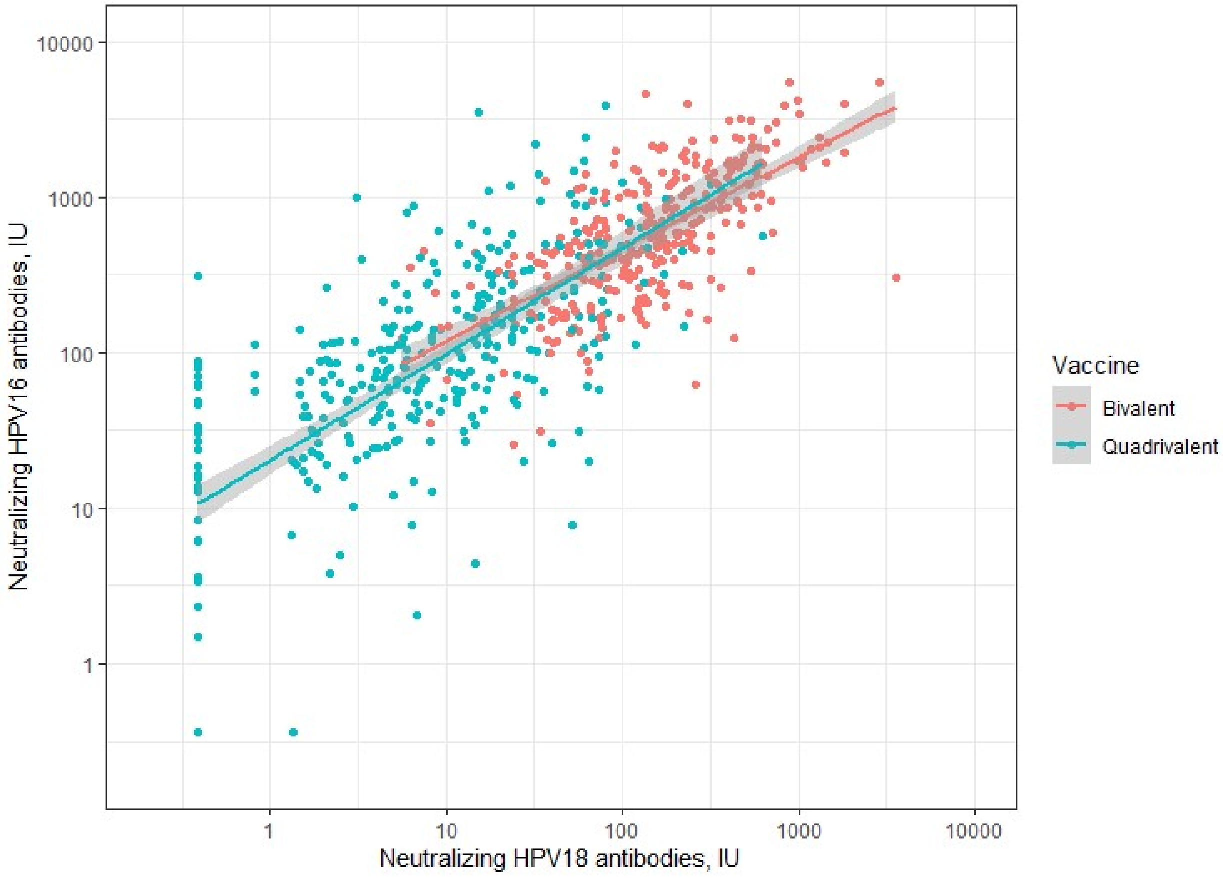

Supplementary Figure 4: Scatter plot of the neutralising HPV6 antibody titres by neutralising HPV18 antibody titre among the vaccine recipients 2-12 years since vaccination with 3 doses of either the bivalent or quadrivalent vaccines. Pearson's correlation coefficient, ρ (among quadrivalent vaccine recipients) = 0.59 (95% confidence intervals, CI, 0.52-0.66), ρ (among bivalent vaccine recipients) =0.17 (95%CI 0.06-0.28).

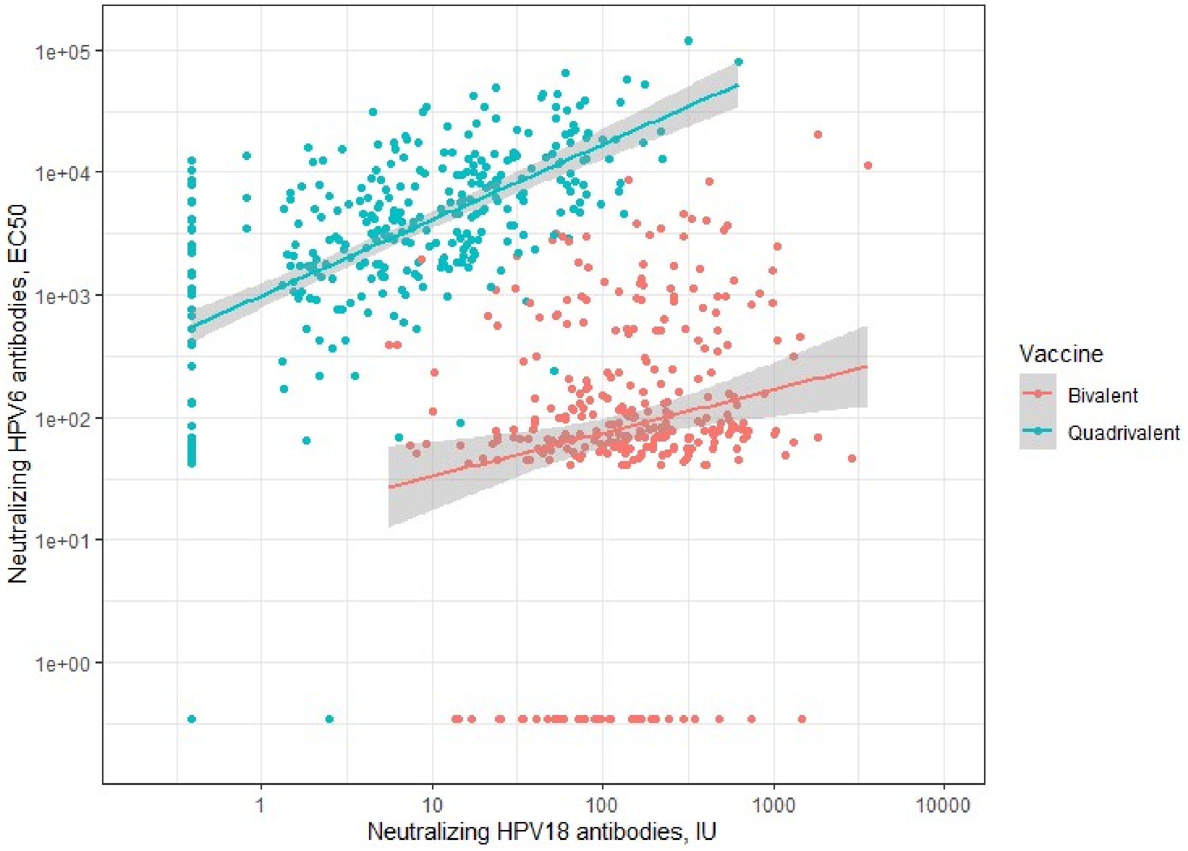

CONSORT 2010 checklist of information to include when reporting a randomised trial*

Section/Topic	Item No	Checklist item	Reported on page No
**Title and abstract**			
	1a	Identification as a randomised trial in the title	na, this study is no longer a randomised trial, as we are only follow-up up the intervention arm
	1b	Structured summary of trial design, methods, results, and conclusions (for specific guidance see CONSORT for abstracts)	page 2, abstract
**Introduction**			
Background and objectives	2a	Scientific background and explanation of rationale	Pages 3-4
	2b	Specific objectives or hypotheses	Page 4
**Methods**			
Trial design	3a	Description of trial design (such as parallel, factorial) including allocation ratio	Page 11
	3b	Important changes to methods after trial commencement (such as eligibility criteria), with reasons	Page 11
Participants	4a	Eligibility criteria for participants	Page 11-12
	4b	Settings and locations where the data were collected	Page 11, references 27 and 28
Interventions	5	The interventions for each group with sufficient details to allow replication, including how and when they were actually administered	Page 11
Outcomes	6a	Completely defined pre-specified primary and secondary outcome measures, including how and when they were assessed	Page 11
	6b	Any changes to trial outcomes after the trial commenced, with reasons	Page 11
Sample size	7a	How sample size was determined	References 27 and 28, page 11-12
	7b	When applicable, explanation of any interim analyses and stopping guidelines	
Randomisation:			
Sequence generation	8a	Method used to generate the random allocation sequence	References 27-28
	8b	Type of randomisation; details of any restriction (such as blocking and block size)	Page 11
Allocation concealment mechanism	9	Mechanism used to implement the random allocation sequence (such as sequentially numbered containers), describing any steps taken to conceal the sequence until interventions were assigned	Page 11
Implementation	10	Who generated the random allocation sequence, who enrolled participants, and who assigned participants to interventions	Pages 11,12, references 27-28
Blinding	11a	If done, who was blinded after assignment to interventions (for example, participants, care providers, those	Page 11

		assessing outcomes) and how	
	11b	If relevant, description of the similarity of interventions	Page 11 and 12
Statistical methods	12a	Statistical methods used to compare groups for primary and secondary outcomes	Page 13,14 and 15
	12b	Methods for additional analyses, such as subgroup analyses and adjusted analyses	Page 13, 14 and 15
**Results**			
Participant flow (a diagram is strongly recommended)	13a	For each group, the numbers of participants who were randomly assigned, received intended treatment, and were analysed for the primary outcome	Page 5, Figure 1
	13b	For each group, losses and exclusions after randomisation, together with reasons	Figure 1
Recruitment	14a	Dates defining the periods of recruitment and follow-up	Page 11
	14b	Why the trial ended or was stopped	References 27, 28,6
Baseline data	15	A table showing baseline demographic and clinical characteristics for each group	Table 1
Numbers analysed	16	For each group, number of participants (denominator) included in each analysis and whether the analysis was by original assigned groups	Tables 1-3
Outcomes and estimation	17a	For each primary and secondary outcome, results for each group, and the estimated effect size and its precision (such as 95% confidence interval)	Tables 2-3
	17b	For binary outcomes, presentation of both absolute and relative effect sizes is recommended	No relative effect size is being measured
Ancillary analyses	18	Results of any other analyses performed, including subgroup analyses and adjusted analyses, distinguishing pre-specified from exploratory	not applicable
Harms	19	All important harms or unintended effects in each group (for specific guidance see CONSORT for harms)	references 27, 28
**Discussion**			
Limitations	20	Trial limitations, addressing sources of potential bias, imprecision, and, if relevant, multiplicity of analyses	Page 10
Generalisability	21	Generalisability (external validity, applicability) of the trial findings	Page 10
Interpretation	22	Interpretation consistent with results, balancing benefits and harms, and considering other relevant evidence	Pages 8-10
**Other information**			
Registration	23	Registration number and name of trial registry	Abstract and methods
Protocol	24	Where the full trial protocol can be accessed, if available	Methods
Funding	25	Sources of funding and other support (such as supply of drugs), role of funders	Page 16

*We strongly recommend reading this statement in conjunction with the CONSORT 2010 Explanation and Elaboration for important clarifications on all the items. If relevant, we also recommend reading CONSORT extensions for cluster randomised trials, non-inferiority and equivalence trials, non-pharmacological treatments, herbal interventions, and pragmatic trials. Additional extensions are forthcoming: for those and for up to date references relevant to this checklist, see www.consort-statement.org.
